# Supplementary material for: Coagulation abnormalities in children with uncorrected congenital heart defects seen at a teaching hospital in a developing country
Source: PLoS One. 2022 Jul 28;17(7):e0263948. doi: 10.1371/journal.pone.0263948 (PMC9333323; doi:10.1371/journal.pone.0263948)
Supplement: S1 Appendix — (DOCX) [file pone.0263948.s001.docx]

- Supporting Information
- STUDY PROFORMA
- BIODATA
- 1. Study ID:……………………
- 2. Age: ……..
- 3. Gender: ………
- 4. Weight: ………. (kg) Length/Height:………. (cm) BMI……Kg/m^2^
- 5. Socio economic classification by Oyedeji
- Level of education Father Mother
- 5. No formal education [ ] [ ]
- 4. Primary [ ] [ ]
- 3. SSCE/GCE Grade 11 teachers training [ ] [ ]
- 2. SSCE/GCE + Professional training [ ] [ ]
- 1. University graduate/equivalent [ ] [ ]
- Parent’s/ Guardian’s Occupation Father Mother
- 1.Senior public servants, professionals, [ ] [ ]
- managers, large-scale traders, businessman
- 2. Intermediate grade, public servants, senior [ ] [ ]
- secondary school teachers
- 3. Junior secondary school teachers, drivers, [ ] [ ]
- Artisans, clerks, typists
- 4. Petty traders, labourers, messengers [ ] [ ]
- 5. Unemployed, full-time housewives, [ ] [ ]
- Students, subsistence farmers

**HISTORY**

- Yes = 1; No = 2
- 1. Any family history of bleeding disorder? [ ]
- 2. Any symptoms/signs of systemic inflammation or infections such as fever? [ ]
- 3. Has the child been on any medications like non-steroidal anti-inflammatory drugs in the last 2 weeks? [ ]
- 4. Is child currently on aspirin, warfarin or anti-platelet medications? [ ]
- 5. Has child been diagnosed of any other illness? [ ]
- 6. If yes (1), please state: ……………………………………………
- 7. Any previous hospital admission in last 1year: [ ]
- 8. If yes (1), how many times and for what:
- **CLINICAL EXAMINATION:**

1. Fever: [ ] (Temperature º C): Cyanosis…………..
2. Capillary Refill Time (CRT):
3. Pulse rate:
4. Respiratory Rate:
5. Oxygen saturation:
6. Hepatomegaly: ………………
7. Cardiac defect

Cyanotic [ ] Acyanotic [ ] No cardiac [ ]

1. Type of cardiac defect:
2. Heart failure class:

- **LABORATORY DATA**
- 1. Prothrombin time
- Test (seconds): …………… Control (seconds): …………
- 2. International Normalized ratio: …………..
- 3. Activated partial thromboplastin time:
- Test (seconds): …………… Control (seconds): …………..
- 4. Haemoglobin level (gm/dl): ……… Haematocrit (%):…………………….
- 5. White blood cell count:…………………………….
- 6. Platelet count (cells/mm^3^): ……..
- 7. D-dimer: ………….. Ref range: …………….
