# Supplementary material for: Coagulation abnormalities in children with uncorrected congenital heart defects seen at a teaching hospital in a developing country
Source: PLoS One. 2022 Jul 28;17(7):e0263948. doi: 10.1371/journal.pone.0263948 (PMC9333323; doi:10.1371/journal.pone.0263948)
Supplement: S1 File — (PDF) [file pone.0263948.s003.pdf]

APPENDIX Ia

ETHICAL APPROVAL

LAGOS UNIVERSITY TEACHING HOSPITAL
HEALTH RESEARCH ETHICS COMMITTEE

PRIVATE MAIL BAG 12003, LAGOS, NIGERIA
e-mail address: luthethics@yahoo.com

Chairman
PROF. N.U. OKUBADEJO
MB. ChB, FMCP

Administrative Secretary
D.J. AKPAN
B.Sc. (Hons) BUS. ADMIN,
MIHSAN

LUTH

Chief Medical Director:
PROF. CHRIS BODE
FMCS (NIG) FWACS

Chairman, Medical Advisory Committee
PROF. O.A. FASANMADE
MBBS, FWACP, FACE, FNSEM

LUTH HREC REGISTRATION NUMBER: NHREC: 19/12/2008a
Office Address: Room 107, 1st Floor, LUTH Administrative Block
Telephone: 234-1-5850737, 5852187, 5852209, 5852158, 5852111

29th August, 2018

NOTICE OF EXPEDITED REVIEW AND APPROVAL

PROJECT TITLE: "COAGULATION ABNORMALITIES IN CHILDREN WITH UNCORRECTED CONGENITAL HEART DEFECTS IN LAGOS UNIVERSITY TEACHING HOSPITAL".
HEALTH RESEARCH COMMITTEE ASSIGNED NO.: ADM/DCST/HREC/APP/2387
NAME OF PRINCIPAL INVESTIGATOR: DR. MAJIYAGBE OMOTOLA OLUFUNMILAYO
ADDRESS OF PRINCIPAL INVESTIGATOR: DEPT. OF PAEDIATRICS, LUTH.
DATE OF RECEIPT OF VALID APPLICATION: 06-07-18

This is to inform you that the research described in the submitted protocol, the consent forms, and all other related materials where relevant have been reviewed and given full approval by the Lagos University Teaching Hospital Health Research Ethics Committee (LUTHHREC).

This approval dates from 29-08-2018 to 29-08-2019. If there is delay in starting the research, please inform the HREC so that the dates of approval can be adjusted accordingly. Note that no participant accrual or activity related to this research may be conducted outside of this dates. All informed consent forms used in this study must carry the HREC assigned number and duration of HREC approval of the study. In multiyear research, endeavor to submit your annual report to the HREC early in order to obtain renewal of your approval and avoid disruption of your research.

The National code for Health Research Ethics requires you to comply with all institutional guidelines, rules and regulations and with the tenets of the code including ensuring that all adverse events are reported promptly to the HREC. No changes are permitted in the research without prior approval by the HREC except in circumstances outlined in the code. The HREC reserves the right to conduct compliance visits to your research site without previous notification.

CHAIRMAN LUTH HEALTH RESEARCH ETHICS COMMITTEE

PROF. N. U. OKUBADEJO
CHAIRMAN, LUTH HEALTH RESEARCH ETHICS COMMITTEE
